# Antibody to Epstein-Barr Virus Deoxyuridine Triphosphate Nucleotidohydrolase and Deoxyribonucleotide Polymerase in a Chronic Fatigue Syndrome Subset

**DOI:** 10.1371/journal.pone.0047891

**Published:** 2012-11-14

**Authors:** A. Martin Lerner, Maria E. Ariza, Marshall Williams, Leonard Jason, Safedin Beqaj, James T. Fitzgerald, Stanley Lemeshow, Ronald Glaser

**Affiliations:** 1 Department of Medicine, Oakland University William Beaumont School of Medicine, Rochester, Michigan, United States of America; 2 Department of Molecular Virology, Immunology and Medical Genetics, The Ohio State University, Columbus, Ohio, United States of America; 3 Center for Community Research, DePaul University, Chicago, Illinois, United States of America; 4 Pathology Inc, Torrance, California, United States of America; 5 Department of Medical Education, University of Michigan School of Medicine, Ann Arbor, Michigan, United States of America; 6 College of Public Health, The Ohio State University, Columbus, Ohio, United States of America; 7 Institute for Behavioral Medicine Research, The Ohio State University, Columbus, Ohio, United States of America; University of Massachusetts Medical School, United States of America

## Abstract

**Background:**

A defined diagnostic panel differentiated patients who had been diagnosed with chronic fatigue syndrome (CFS), based upon Fukuda/Carruthers criteria. This diagnostic panel identified an Epstein-Barr virus (EBV) subset of patients (6), excluding for the first time other similar “clinical” conditions such as cytomegalovirus (CMV), human herpesvirus 6 (HHV6), babesiosis, ehrlichiosis, borreliosis, *Mycoplasma pneumoniae*, *Chlamydia pneumoniae*, and adult rheumatic fever, which may be mistakenly called CFS. CFS patients were treated with valacyclovir (14.3 mg/kg q6h) for ≥12 months. Each patient improved, based upon the Functional Activity Appraisal: Energy Index Score Healthcare Worker Assessment (EIPS), which is a validated (FSS-9), item scale with high degree of internal consistency measured by Cronbach's alpha.

**Methods:**

Antibody to EBV viral capsid antigen (VCA) IgM, EBV Diffuse Early Antigen EA(D), and neutralizing antibodies against EBV-encoded DNA polymerase and EBV-encoded dUTPase were assayed serially approximately every three months for 13–16 months from sera obtained from patients with CFS (6) and from sera obtained from twenty patients who had no history of CFS.

**Results:**

Antibodies to EBV EA(D) and neutralizing antibodies against the encoded-proteins EBV DNA polymerase and deoxyuridine triphosphate nucleotidohydrolase (dUTPase) were present in the EBV subset CFS patients. Of the sera samples obtained from patients with CFS 93.9% were positive for EA(D), while 31.6% of the control patients were positive for EBV EA(D). Serum samples were positive for neutralizing antibodies against the EBV-encoded dUTPase (23/52; 44.2%) and DNA polymerase (41/52; 78.8%) in EBV subset CFS patients, but negative in sera of controls.

**Conclusions:**

There is prolonged elevated antibody level against the encoded proteins EBV dUTPase and EBV DNA polymerase in a subset of CFS patients, suggesting that this antibody panel could be used to identify these patients, if these preliminary findings are corroborated by studies with a larger number of EBV subset CFS patients.

## Background

One major problem for investigators studying CFS is the heterogeneity of the population united by common life-altering symptoms without scientific laboratory confirmation. With Fukuda/Carruthers criteria and a systematic review of 142 chronic fatigue syndrome (CFS) patients [1]–[6], two groups of CFS patients were defined. To achieve a homogenous CFS population, Group A CFS patients have elevated serum IgG antibody to the herpesviruses Epstein-Barr virus (EBV), alone or along with cytomegalovirus (CMV) and human herpesvirus 6 (HHV6); no other co-infections are identified. Group B CFS patients have similar elevated herpesvirus antibody titers, plus serologic evidence of other co-infections, including tick-borne *Borrelia burgdorferi*, *Anaplasma phagocytophilia*, *Babesia microti*: *Mycoplasma pneumoniae*: *Chlamydia pneumoniae* infection or adult rheumatic fever. One hundred and six group A CFS patients were followed in this systematic review from this center (2001 – 2007), and treated for ≥12 months with subset directed valacyclovir, for EBV subset, or valganciclovir, for HCMV and HHV6 subsets [2], [7], [8]. The data include over 5000 patient visits and 35,000 data entries. Seventy-nine (74.5%) of the Group A patients recovered based upon their functional activity appraisals: energy index score healthcare worker assessment [9] and their ability to resume a 40-hour workweek and normal social activities (p<0.0001) [2].

An evidence-based test for the diagnosis of CFS remains elusive. Glaser, Williams and Lerner hypothesize CFS may be related to abortive lytic replication of EBV in the absence of a DNAemia, or IgM antibody to virus structural protein [10]–[16]. Glaser, Williams and co-workers found that the early EBV encoded protein deoxyuridine triphosphate nucleotidohydrolase (dUTPase) induced leukocytes to synthesize several proinflammatory cytokines in vitro, which are similarly elevated in some CFS patients. This EBV encoded dUTPase also induced immune changes and sickness in mice [13]. Similar evidence for viral induced immune dysregulation and changes in intracellular perforins and granzymes were found in CFS patients [17].

Valacyclovir and valganciclovir are phosphorylated to the triphosphate derivatives by virus encoded thymidine kinases/phosphotransferases as well as cellular enzymes, where they act as alternative substrates for the herpesviruses encoded DNA polymerases and inhibit viral DNA replication by preventing DNA chain elongation. Since valacyclovir and valganciclovir do not inhibit the synthesis of early herpesvirus proteins, thus inducing a type of abortive-lytic replication, we suggested that new herpesvirus host cell recruitment is interrupted in the CFS patients treated with valacyclovir/valganciclovir who recovered their health [16]. It is possible that one or more herpesvirus early proteins may be important to CFS pathophysiology. We earlier reported elevated HCMV IgM serum antibody titers to early proteins p52 (UL44) and CM2 (UL 44 – UL 57) in 61 CMV subset CFS patients. These early CMV encoded elevated serum antibody titers were not present in a comparison group of normal patients [18]. We also discovered elevated serum antibody titers to EBV EA(D) in 86 of the 106 (81%) CFS patients with group A CFS [2].

The EBV encoded early viral proteins, dUTPase and DNA polymerase are enzymes involved in EBV lytic DNA replication. We now report the significant repetitive presence of positive serum antibodies to the EBV encoded dUTPase and DNA polymerase in 6 Group A EBV subset CFS patients [2]. During 13 – 16 consecutive months 2003 – 2007, elevated serum neutralizing antibody was present to EBV encoded dUTPase in 23/52 serum samples (44.2%) and to the EBV DNA encoded polymerase (41/52 assays, 78.8%) for over 400 days during treatment with valacyclovir. Comparison group tests for neutralizing antibody to the EBV-encoded dUTPase and DNA polymerase from 20 random age-sex matched persons having routine blood specimens at a commercial laboratory were negative (Presented in part at the 10th IACFS Conference for Physicians and Healthcare Professionals Translating Evidence into Practice, September 2011, Ottawa, Canada as a poster). Elevated serum neutralizing antibody to EBV encoded dUTPase and EBV DNA polymerase suggests that incomplete or abortive lytic replication has taken place, which is expected because of the mode of action of the antiviral agent used to treat these patients. The preliminary data demonstrate that there is a prolonged elevated antibody level against a subset of patients with CFS, suggesting that this antibody panel could be used to identify such patients.

## Methods

### Ethics Statement

This study was approved by the Human Investigation Committee of William Beaumont Hospital. The requirement for consent was waived by the IRB/ethics committee because samples were archived and patient identification was not made.

### CFS Patients (Figure 1)

The six CFS patients were identified as; Group A EBV subset (five patients), and Group B (one patient) who was co-infected with *Borrelia burgdorferi* [*2*]. The single CFS Group B patient had a positive western blot IgM test for *Borrelia burgdorferi*. CFS patients were receiving valacyclovir. CFS patients' numbers 1,2,3,4 and 6 had negative ELISA CMV IgG serum titers. CFS patient number 5's initial serum CMV IgG titer was 71. CMV IgM titers were negative in all patients [2]. HHV6 serum titers were done by LabCorp (Dublin, Ohio) [2]. HHV6 IgG values were: patient number 2, 80; patient number 3, 40; patient number 4, 10; patient number 6, 40; and patient numbers 1 and 5, unknown [2]. HHV6 IgM titers were negative in patients 2,3,4 and 6.

**Figure 1:**
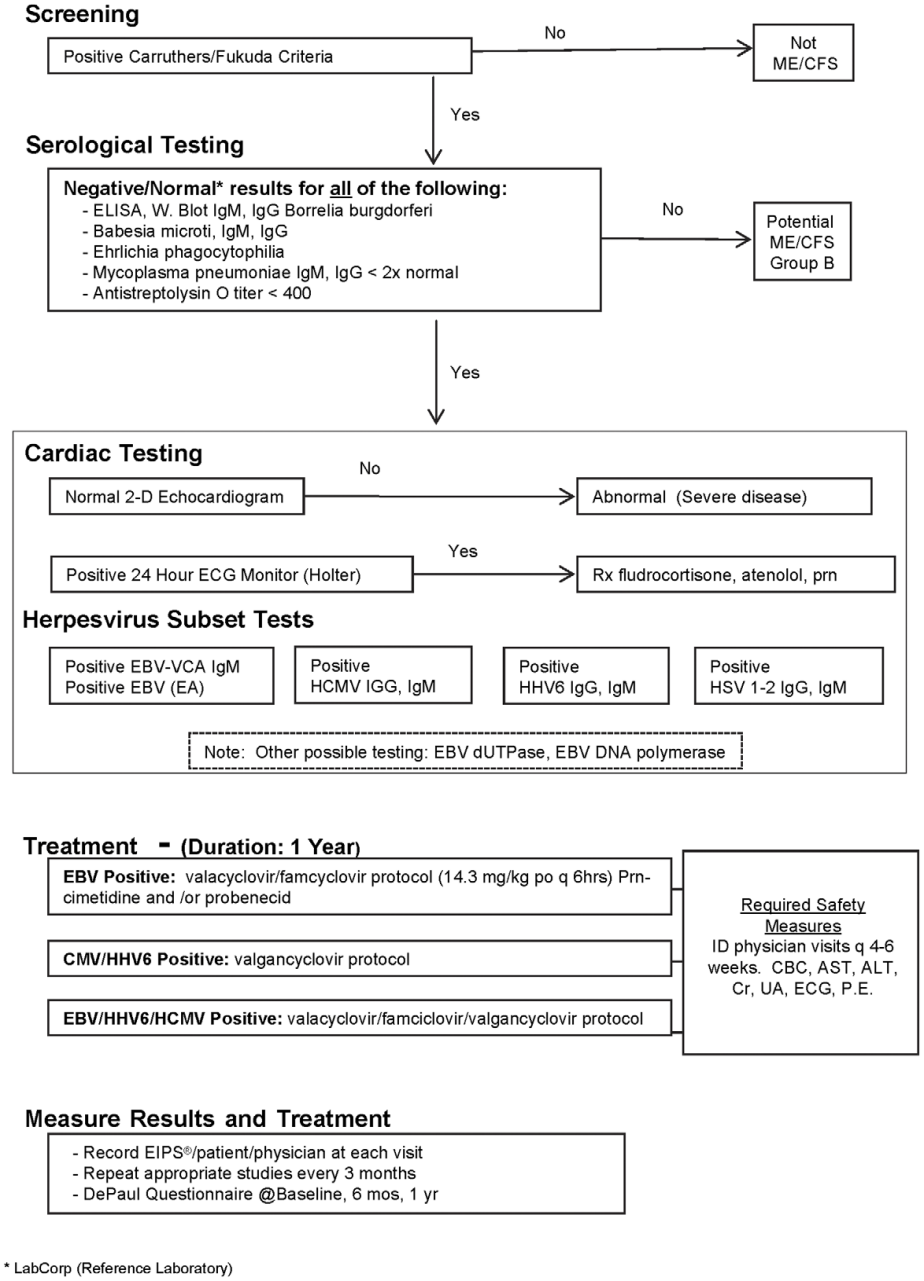
This diagnostic decision tree identifies Group A CFS patients.

### Comparison Group

Blood samples were taken (6/28/11) from unknown persons having health related studies at a commercial laboratory. Age and sex of the comparison group were selected to be similar to the CFS group.

### Antibody to EBV VCA IgM, and the VCA p18 peptide was measured

The p18 peptide is a defined VCA-specific marker protein utilized in the ETI-EBV-M reverse assay (DiaSorin, Inc., Stillwater, MN, USA). It consists of 56 amino acids of BFRF encoded VCA and contains immunodominant epitopes. The ETI-EBV-M reverse kit utilizes the enzyme-linked immunosorbent assay (ELISA) based on the antibody capture technique. The absorbance of the solution measured at 450nm is related to the concentration of IgM to EBV VCA present in the reaction solution [1]. A value of <20 is considered negative.

### EBV-IgG EA(D)

The ETI-EA-G kit (DiaSorin) for quantitative detection of IgG antibodies to the EBV EBV-EA(D) was used. Diluted serum was incubated with recombinant EA(D) peptide bound to the solid surface of a micro titer well. The ETI-EA-G assay uses an EA(D) 47 KD recombinant polypeptide. The absorbance of the solution, measured at 450 nm is proportional to the concentration of IgG antibodies to EBV EA(D) present in the reaction solution [1]. A value of <20 is considered negative.

### CMV ELISA

ELISA testing for CMV IgG and CMV IgM was performed using ELISA kits from DiaSorin. The CMV IgG kit contains purified CMV strain AD-169 antigen-coated wells. The CMV IgM ELISA is a microcapture assay with wells coated with anti-human IgM antibody to the same strain AD-169. Sera were diluted 1∶10 and incubated for one hour at 37°C. The wells were washed three times in washing buffer and bound HRP label was detected with 3,3′ 5.5 tetramethyl benzidine as substrate for 30 minutes in the dark, after which the color reaction was stopped by the addition of stop solution as recommended by the manufacturer's manual. The absorbance was measured at 450/650 nm using Biotech reader (Biotech Clinical Laboratories, Inc., Farmington MI, USA) [18]. A value of <18 is considered negative.

### Neutralization assays (DNA polymerase and dUTPase) were performed as previously described [13], [15]

Briefly, 5 µl of human serum were mixed with 5 µl of either purified EBV-encoded dUTPase (3–5 units of enzyme) or an extract from TPA/sodium butyrate induced Raji cells (for EBV-encoded DNA polymerase) for 30 min at room temperature prior to assaying for enzymatic activity. EBV-encoded DNA polymerase and dUTPase activity were determined as described previously [14], [15]. Raji cells were induced by treatment with TPA and sodium butyrate for 48 hrs. Cells (10^6–8^) were harvested, resuspended in 1 ml of extraction buffer (50 mM Tris-HCl, pH 8.0 2 mM ATP, 0.2 M KCl, 3 mM dithiothreitol, 2 mM MgCl2 0.2 mM phenylmethylsulfonylfluoride and 10% (v/v) glycerol, lysed by sonication and centrifuged at 14,000×g for 5 min. The resulting supernatant was employed for the EBV-encoded DNA polymerase assay. Purified EBV-encode dUTPase was obtained as we have described [13].

For positive controls, assays were performed in the presence of human serum that lacks detectable antibody to the EBV encoded dUTPase and DNA polymerase; negative controls were also performed in the absence of the enzyme preparation. A unit of EBV-encoded dUTPase activity was defined as the amount of enzyme required to convert 1nmole of dUTPase to dUMP and pyropohosphate/min/ml of enzyme at 37 C [13], while a unit of EBV-encoded DNA polymerase activity was defined as the amount of enzyme required to incorporate 1 pmole of dTTP into activated calf thymus DNA/min/ml at 37 C [14]. Units of enzymatic activity neutralized per ml of serum were obtained as follows: (U_control_ – U_serum_). Serum with neutralizing units greater than or equal to two standard deviations from the control were considered “positive” for dUTPase or DNA polymerase neutralizing antibodies. Quantitative titers of antibody to EBV dUTPase and EBV DNA polymerase were assayed.

The following tests were performed by LabCorp (Dublin, Ohio) on the 6 CFS patients (Group A, 5 patients; Group B, 1 patient) in order to determine if the subjects were co-infected with other infectious agents [2].

#### Lyme, Western Blot and ELISA, serum – IgG and IgM. Method

Antigen – whole-cell proteins were extracted from *B. burgdorferi* strain B31, resolved by polyacrylamide gel electrophoresis into individual antigen bands and then transferred to nitrocellulose strips for blotting.

#### Babesia microti Antibody Panel – IgG and IgM

Method – IFA. Antigen – the substrate for the IFA was guinea pig or hamster erythrocytes infected with *Babesia microti* organisms and then fixed onto microscope slides. Upon interaction with human sera containing anti-Babesia antibodies and the appropriate conjugate, infected cells fluoresce.

#### Ehrlichia Ab panel “(Granulocytic and Monocytic/Anaplasma phagocytophilia)” – IgG and IgM

Method: IFA. Antigen: is either inactivated HGE or HME.

#### Mycoplasma pneumoniae Antibodies – IgG and IgM

Method: EIA. Antigen: *Mycoplasma pneumoniae* FH antigen.

#### Antistreptolysin 0 Ab

Method: Latex immunoturbidimetry. Human Antistreptolysin 0 antibodies agglutinate with latex particles coated with streptolysin 0 antigens. The precipate is determined turbidimetrically at 552 nm.

### Statistical Analyses

Blood test results for EA(D), dUTPase, and DNA polymerase were scored as “positive” or “negative”. For each test, two comparisons were completed; 1) the CFS patient group's initial (baseline) test scores were compared to the control group's scores, and 2) the CFS patient group's last recorded test score (the frequency an individual was tested ranged from 7 to 10) were compared to the control group's scores. No analysis was conducted for the VCA, IGM measure as all scores were negative.

Fisher's exact test was used to compare the two groups. A Bonferroni adjustment for multiple statistical tests determined the appropriate alpha level for a two-tailed test to be p<0.01.

## Results

### Demographics

The six patients (5 women) with CFS were EBV subset of CFS patients, with 5 patients in the Group A subset and one patient (#2) in Group B. CFS patients were 37 – 59 years of age. Serum samples were taken at intervals during valacyclovir therapy from 3/5/02 to 11/14/03. There were 7 to 10 sera samples from each of the 6 CFS patients. Five of the 6 CFS patients were Group A (no co-infections); one patient had a co-infection with *Borrelia burgdorferi* (patient no. 2). Initial EIPS values were 3.5 – 5.0, meaning that patients could be out of bed only 3 to 4 hours a day, and required daily naps to complete each day. One CFS patient was able to struggle to complete a sedentary working day. This male member of the CFS group did not meet criteria for CFS at baseline (EIPS, 6). He struggled at baseline to maintain his sedentary working day, required a daytime nap, and could no longer do any exercise without marked syncope and worsening fatigue. One year later the final EIPS values were 7 – 8, for the 5 Group A CFS patients, meaning that patients could now live normal lives. The single Group B CFS patient's final EIPS value increased from a baseline of 3.5 to 5, but this woman still met international criteria diagnosis of CFS [4], [5]. The EIPS is a validated (FSS-9 item scale with high degree of internal consistency measured by Cronbach's alpha) Functional Activity Appraisal: Energy Index Score Healthcare Worker Appraisal [9].

The mean age of the comparison group was 48.7 years (36 – 59). Fifteen of 19 (78.9%) persons were women.

### EBV Encoded Gene Products

#### EBV, VCA IgM

VCA IgM titers were performed on 49 sera from CFS subjects, as well as the twenty comparison samples. All were negative. The presence of a positive serum EBV VCA IgM indicates lytic virus replication. Approximately 15% of EBV subset CFS patients have positive serum EBV VCA IgM titers [19]. Virtually everyone, EBV subset CFS patients and healthy controls have positive serum EBV VCA IgG titers indicating past infection. Therefore, we did not assay EBV VCA IgG titers in CFS patients or in controls.

#### EBV, EA (D)

Forty-nine EA(D) serum samples from patients with CFS were examined for antibodies against EA (D). All were positive except for three serum samples obtained from CFS patient number 6. Mean EBV, EA(D) titers (by patient) were: 54 (patient 1); 123 (patient 2); 63 (patient 3); 128 (patient 4); 49 (patient 5); and 27 (patient 6), negative < 20. EBV, EA(D) positive; mean cumulative antibody titers of the 6 patients was 74. Conversely, only 6 of the sera samples of the nineteen tested from the comparison group were positive (31.6%) for antibody against EA (D) with a cumulative mean of 22. The significance of difference in presence of encoded proteins EBV, EA (D) between EBV subset patients and controls according to first sera tests by Fisher's Exact test (two-sided P value) is 0.0109, considered significant. There was a significant row and column association (p = 0.01) for both the first and last CFS patient values of the series for the occurrence of the EBV, EA(D) when compared to the comparison group values. Therefore, the occurrence of EBV EA(D) serum antibody is a significant difference between EBV subset CFS and controls.

#### EBV-encoded dUTPase

Quantitative assays to determine the units of EBV-encoded dUTPase neutralized/ml of serum demonstrated a mean of 5 units neutralized/ml of serum from patients with CFS compared to 2 units neutralized/ml of serum from controls. Three of 10 (30%), CFS (patient 1); 5 of 7 (71.4%) CFS (patient 2); 3 of 10 (30%) CFS (patient 3); 8 of 10 (80%) CFS (patient 4); 3 of 8 (37.5%) CFS (patient 5); 1 of 7 (14%) CFS (patient 6) were positive for elevated serum antibody levels to EBV dUTPase. Twenty-three of the 52 (44%) serum samples were positive for neutralizing antibody against the EBV dUTPase.

Fisher exact tests were done for first sera compared to that of the comparison group as well as for the last sera compared to the comparison/control group. The difference between the last CFS patient measure and the control group values did not quite achieve statistical significance (p = 0.074). For the first EBV dUTPase assay, the p value is significant (p<0.01). (Table 1).

**Table 1:** Significance of Differences in Presence of EBV Encoded Protein dUTPase Between EBV Subset Patients and Controls.

	First dUTPase Assay		Last dUTPase ssay	
	Target Group	Control Group	Target Group	Control Group
Positive	6	0	2	0
Negative	0	20	6	20
Total	6	20	8	20

#### EBV- encoded DNA polymerase

Quantitative assays to determine the units of EBV-encoded DNA polymerase neutralized/ml of serum demonstrated that a mean of 60 units neutralized/ml of serum from patients with CFS compared to 16 units neutralized/ml of serum from controls. Eight of 10 serum samples (80%) of CFS (patient 1); 4 of 7 (57.1%) CFS (patient 2); 7 of 10 (70%) CFS (patient 3); 9 of 10 (90%) CFS (patient 4); 7 of 8 (88%) CFS (patient 5); and 6 of 7 (85.7%) (patient 6) had antibody titers to EBV encoded DNA polymerase. Forty-one of the 52 (78.8%) CFS samples from CFS patients were positive for antibodies against the EBV-encoded DNA polymerase. For both the first and last recorded values, the differences for EBV DNA polymerase tests between CFS patients and controls are significant (p<0.01 for both comparisons). (Table 2).

**Table 2:** Significance of Differences in Presence of EBV Encoded Protein DNA Polymerase Between EBV Subset CFS Patients and Controls.

	First DNA Polymerase Assay		Last DNA Polymerase Assay	
	Target Group	Control Group	Target Group	Control Group
Positive	3	0	5	0
Negative	3	20	1	20
Total	6	20	6	20

**Figure 2:**
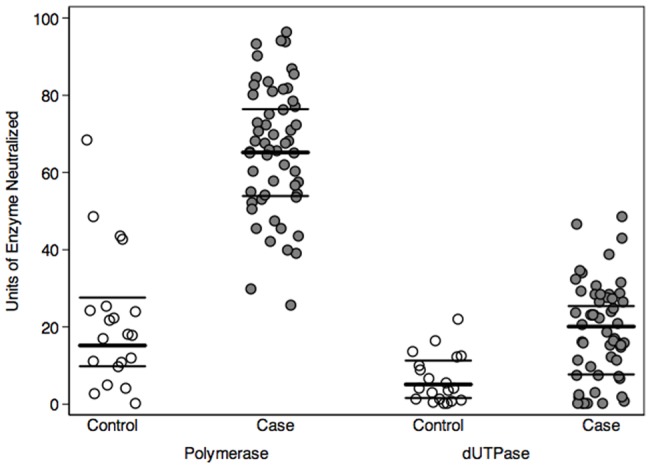
Neutralizing antibodies against EBV-encoded DNA polymerase and dUTPase in EBV subset CFS patients and controls. Neutralizing Antibodies Against EBV-encoded DNA polymerase (units/ml) and dUTPase (units/ml×10) in patients according to control versus patients with CFS who were treated with valacyclovir.

**Figure 3:**
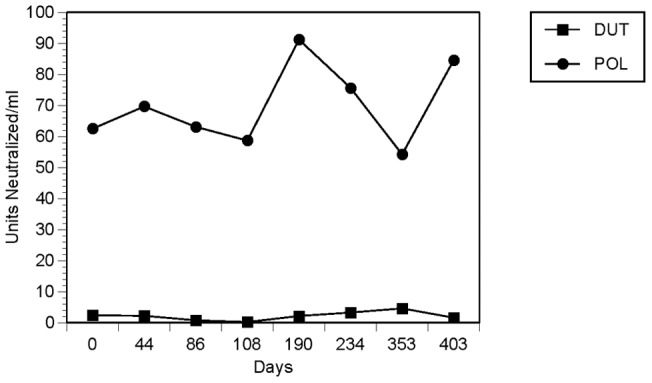
Antibody response to EBV encoded proteins DNA polymerase and dUTPase during treatment with valacyclovir. Duration of Antibody Response to EBV Encoded Proteins EBV DNA Polymerase and EBV dUTPase During Treatment with Valacyclovir.

As shown in Figure 2, GroupA/B patients with CFS exhibited a greater humoral response to the EBV-encoded dUTPase and DNA polymerase as determined by a statistically significant increase in neutralizing antibodies against these EBV encoded enzymes when compared to the comparison group. The increase in neutralizing antibodies in the CFS patients against the EBV-encoded dUTPase and DNA polymerase was sustained over the course of the patient's illness. A typical example is shown for patient 5 in Figure 3.

## Conclusions

The EBV, a DNA human tumor virus, encodes for six viral enzymes that are part of the early antigen (EA) complex. These proteins are synthesized prior to EBV DNA replication. Such proteins are classified as early proteins [20]–[25]. Work from our laboratory has focused on unique antibody patterns to EBV-encoded enzymes, e.g. DNase, DNA polymerase and dUTPase. We found that patients with nasopharyngeal carcinoma (NPC) have antibodies to EBV-encoded DNase [26]. Out of 101 serum samples from normal EBV seropositive patients (medical students), none were positive for antibody to EBV-encoded DNase, whereas 94% of 49 serum samples from NPC patients were positive for antibody to the EBV-encoded DNase [27].

NPC patients may also make antibody to the EBV TK [28]. Patients with infectious mononucleosis (IM), chronic active EBV infection, and patients infected with HIV have elevated antibody titers to EBV-encoded dUTPase [12]. While the unique antibody patterns to these EBV proteins (or other herpes virus proteins) and other EBV-encoded proteins have been found to be clinically useful [1], [28]–[30], the underlying factor(s) that produce these antibody patterns to EBV proteins and the role these proteins might play in the pathophysiology of EBV-associated disease, separate from their role in the replication of the virus, is still being explored. There have not been many studies to determine whether EBV-encoded proteins expressed during lytic and/or abortive-lytic replication have immunoregulatory properties. This is due in part to the lack of an *in vitro* cell system that is fully permissive for EBV lytic infection. However, it is well documented that antibodies to various EBV encoded proteins, which are involved with lytic replication of the virus, are produced during EBV infections. Antibodies to EBV encoded proteins occur in patients with IM, Burkitt lymphoma (BL), NPC and CFS and the presence of these antibodies have clinical significance [31].

It was proposed that one or more of the EBV early proteins, which are synthesized after the latent virus is reactivated, alone or in combination with other EBV-encoded (or other latent herpesvirus encoded) proteins could play a role in the pathophysiology of EBV-associated disease [32], [33]. We published the first evidence that the EBV-encoded dUTPase is able to induce immune dysregulation *in vitro* as demonstrated by its effect on the replication of PBMCS and the production of several different proinflammatory cytokines including IL-1β, TNF-α, IL-6, IL-8 and IL-10 [13]. We have also shown that the EBV dUTPase can induce immune dysregulation and sickness behavior in mice [11]. We hypothesize; that the immune dysregulation induced by EBV-encoded dUTPase (and perhaps other herpesvirus encoded proteins yet to be identified) play a role in the pathophysiology of EBV associated disease. The availability of these EBV early proteins to induce an antibody response could result from the lysis of cells in which the latent EBV genome was fully reactivated. It is also possible that early proteins like the EBV-encoded dUTPase can be released by cells undergoing abortive reactivation through exosomal release or through apoptosis of the infected cell [34]. Whether the early proteins synthesized by other herpesviruses can induce immune dysregulation needs to be explored. However, the data from our study [13] and others [35]–[37] show that at least some of the viral encoded proteins can produce changes in both humoral and cellular immunity separate from their roles in virus replication/latency.

In this study we demonstrate that there is a statistically significant increase in antibody levels to EBV EA-D complex, EBV-encoded dUTPase and EBV-encoded DNA polymerase in repetitive longitudinal serum samples obtained from six CFS Group A EBV subset patients studied over a consecutive period of 13–16 months who were treated with valacyclovir when compared to a control group. Particularly antibodies to the EBV-encoded protein DNA polymerase here separate the EBV subset CFS patients from the comparison group patients. The CFS EBV subset patients identified in this study are a distinct laboratory based group whose identification was made possible for the first time by the development of a unique diagnostic panel, which selected the study patients. Other previous CFS studies did not have available this diagnostic panel [2].

In a double-blinded placebo-controlled study Fluge, et al [38] have recently reported transient clinical improvement of CFS symptoms in patients meeting Fukuda/Carruthers criteria for diagnosis using two intravenous infusions of the monoclonal B-lymphocyte depleting anti-CD20 antibody (500 mg/m2) Rituximab given two weeks apart. There was a clinical benefit to 10 of 15 CFS patients after the Rituximab infusions. As pointed out by Fluge et al [38] the improved clinical response of these patients may be at least in part due to elimination of B-lymphotrophic viruses which supports our previous studies [2] as well as those of Kogelnik which are consistent with abortive lytic replication [7]. Similarly, Strayer, et. al. reported improvement in exercise tolerance and reduction in CFS symptoms with a phase III IV rintatolimod versus placebo randomized placebo-controlled trial (p = 0.04) [39]. Rintatolimod is an activating ligand (dsRNA) for TLR3, which is a first line of defense mechanism in the induction of innate immunity [39]. EBERs are nonpolyadenylated and non-coding RNA expressed in cells latently infected with EBV have also been reported to activate TLR3 [40]. Both the Fluge [38] and Strayer studies [39] are consistent with the herpesvirus' CFS paradigm proposed by our group [10], [16] and supported by the presence of elevated serum antibody to encoded proteins EBV DNA polymerase and EBV dUTPase reported here. CFS patients have impaired NK cell function and numbers [17]. These NK changes may be either primary genetic, or due to herpesvirus abortive lytic replication, we describe.

We note that we [15] and Natelson, Moul and Jenkins, et. al. [41] reported elevated serum antibody titers to EBV DNA polymerase in some patients with CFS without the critical definition of Group A EBV subset CFS of this report [2]. Neither earlier study treated CFS patients with valacyclovir. Patients with Group A CFS with subsets CMV or HHV6 do not respond to valacyclovir [2]. Likewise, Group B CFS patients with unrecognized co-infections do not respond to valacyclovir [2]. What we find remarkable when comparing our new data to the data we published in our 1988 paper [15] is the consistency of the antibody patterns to the EBV encoded proteins with the antibody patterns in this study. Of interest is the fact that there is evidence that CFS patients may be at a higher risk for lymphoma. In a previous study from our laboratory on CFS and EBV encoded DNAse and DNA polymerase, three of six CFS patients studied who had elevated anti-EBV enzyme antibody levels developed fatal lymphoma [15]. A study by Paul Levine and co-workers support the association [42]. A recent report confirmed that there is a relationship of CFS with malignant disease [43]. The role that EBV encoded enzymes play in the pathophysiology of EBV associated disease, including CFS infection is an area of research that may be important in elucidating the etiology and treatment of CFS and some lymphoid tumors
